# Association of some psychosocial factors with anthropometric measures in nationally representative sample of Iranian adolescents: the CASPIAN-III study

**DOI:** 10.1186/s40200-016-0237-7

**Published:** 2016-06-01

**Authors:** Fereshteh Baygi, Roya Kelishadi, Mostafa Qorbani, Fatemeh Mohammadi, Mohammad Esmaeil Motlagh, Gelayol Ardalan, Morteza Mansourian, Seyed Masoud Arzaghi, Hamid Asayesh, Ramin Heshmat

**Affiliations:** 1 Occupation Health Research Center, Iran University of Medical Sciences, Tehran, Iran; 2 Child Growth and Development Research Center, Research Institute for Primordial Prevention of Non-communicable Diseases, Isfahan University of Medical Sciences, Isfahan, Iran; 3 Dietary Supplements and Probiotics Research Center, Alborz University of Medical Sciences, Karaj, Iran; 4 Chronic Diseases Research Center, Endocrinology and Metabolism Population Sciences Institute, Tehran University of Medical Sciences, Tehran, Iran; 5 Department of Food and Nutrition Policy and Planning Research, National Nutrition and Food Technology Research Institute, Faculty of Nutrition Sciences and Food Technology, Shahid Beheshti University of Medical Sciences, Tehran, Iran; 6 Pediatrics Department, Ahvaz Jundishapur University of Medical Sciences, Ahvaz, Iran; 7 Department of Health Education and Promotion, Iran University of Medical Sciences, Tehran, Iran; 8 Elderly Health Research Center, Endocrinology and Metabolism Population Sciences Institute, Tehran University of Medical Sciences, Tehran, Iran; 9 Department of Medical Emergencies, Qom University of Medical Sciences, Qom, Iran

**Keywords:** Overweight, Obesity, Adolescent, Psychosocial factors

## Abstract

**Background:**

During the last two decades, adolescent obesity has increased in western countries. In Iran-as a developing country- the prevalence of obesity is raised among youngsters as well. This study conducted to identify an association of adolescents’ loneliness, self-confidence and relationship with others in home and school environment with their weight status.

**Methods:**

In this cross-sectional national survey, 5682 students aged 10–18 years from urban and rural districts of 27 provinces of Iran were selected via stratified multi-stage sampling method. Data on psychological problems of students was gathered through a questionnaire. Height, weight, and waist circumferences were measured according to standard protocols. Body mass index (BMI) and waist- to-height ratio was calculated.

**Results:**

Boys which did not have best friends, spend time with their friends after school or get acceptance from them, had higher BMI than others. Only girls who did not spend time with their friends had higher BMI (19.48 ± 4.28) vs. (19.09 ± 3.92) and WC (71.04 ± 21.29) vs. (69.15 ± 17.43) than others, *P* < 0.05. In both sexes, adolescents who had sense of pressure about doing homework or had difficulties in relationship with their parents had higher BMI and WC values. Girls who reported being victim of violent behaviors (being bullied), had lower BMI compared to others. Risk of being overweight and obese, but not abdominal obese was statistically higher in adolescents not having close friends (OR = 1.81, CI: 1.11–2.95). Lack of self-confidence increased only the risk of obesity in teens (OR = 1033, CI: 1.09–1.64).

**Conclusions:**

Our findings suggest that strategies for prevention of overweight and obesity in adolescent should be taking into account a deeper knowledge of psychosocial issues due to be able to design more effective programs for treating overweight teens.

## Background

During last two decades, adolescent obesity has increased in western countries [1]. In Iran-as a developing country- the prevalence of obesity is raised among youngsters as well [2]. The overall prevalence of obesity and overweight remained relatively constant during 2000 to 2004 and 2005 to 2010 and are estimated to be about 5.1 % (95 % confidence interval [CI], 4.4–5.8) and 10.8 % (95 % CI, 10.2–11.4), respectively [3]. Overweight children and adolescents may experience some psychosocial problems such as depression, social isolation, and poor self-confidence, dissatisfaction with body-image, reduced quality of life, family disharmony and trouble with teachers [4]. Current epidemiological data show that up to 20 % of children and adolescents suffer from a mental health problem and up to 50 % of these problems onset in adolescence. In the other words, adolescence is a substantial period for development of mental disorders such as depression [5]. Most obese adolescents remain obese as adults, so this age group is an important group for designing preventive programs addressing issues of health promotion, (e.g., body satisfaction, sociability, diet and sedentary behaviors). Overweight and obese adolescents with good body image are more likely to control their weight [6] whereas adolescents with poor body image are at risk for weight gain [7]. Social engagement may affect adolescent body satisfaction [8]. Engaging with friends and a social resource may increase socio-emotional maturity and self-confidence in adolescents [9]. In depressed adolescents the risk for development and persistence of obesity during adolescence are increased [10]. Depression can stimulate neuro-endocrine responses. Activation of Hypothalamic-pituitary axis and sympathetic nervous system may induce abdominal adiposity, insulin resistance and metabolic syndrome through increasing cortisol production [11]. Positive association between some psychological factors (e.g. depression in adolescence) and BMI during adulthood was observed [1]. Clinicians need to identify and help them to address the psychosocial factors that may be contributing to their child’s or adolescent’s obesity. Affected individuals may suffer from depression, low self-esteem, bullying, and weight bias, experiences that can make achieving desired health outcomes more difficult [12]. Most studies assessed the shared biological and psychosocial determinants of obesity-due to cross-sectional design- they are unable to detect causal relationships. Whether factors like low self-esteem and depression lead to obesity or whether obesity causes them, remains unclear [1]. Therefore, studies to determine psychosocial factors that may distinguish obese and overweight adolescents from their peers are needed.

### Objectives

This study is conducted to evaluate association of anthropometric measures with some psychosocial factors in nationally representative sample of Iranian adolescents.

## Methods

This national level cross-sectional study was performed in 2009–2010 as the third survey of the school-based surveillance system entitled **C**hildhood and **A**dolescence **S**urveillance and **P**revent**I**on of **A**dult **N**on communicable disease (CASPIAN-III) Study in Iran. It was a multicentre study performed among 5682 school students (96 % participation rate), aged 10–18 years, living in urban and rural areas of 27 provinces in the Iran. Details on the study protocol have been described before [13] and here we report it in brief. The sample size was calculated according to the multistage cluster sampling method. The maximum sample size that could give a optimal estimate of all risk factors of interest was selected. Thus, the sample size was calculated as 210 subjects in each province. A total of 21 clusters of 10 subjects in each of the provinces and a total of 5670 students and an equal number of their parents were selected from 27 provinces. Children and adolescents were selected by multistage random cluster sampling from urban and rural areas of each provinces of Iran.

Required approvals have been documented from ethical committees of Isfahan University of Medical sciences (IUMS), Tehran University of Medical sciences (TUMS), and other relevant regulatory organizations at national and provincial levels. The Data and Safety Monitoring Board of the project closely supervised the quality control and quality assurance of the survey at the national level.

All procedure of data gathering and data managing were conducted carefully anonymously under security considering.

The project team obtained written informed consent from parents and oral assent from students. They selected school students by multistage random cluster sampling. Schools were stratified according to location (urban or rural), and the socioeconomic characteristics of their catchment area, taking into consideration the proportion of the different types of schools (public or private) to avoid socioeconomic bias.

Data on psychological problems of students was gathered through a questionnaire based on the standard protocols of WHO global school-based student health survey with participation of trained teams of expert health care providers. The validity and reliability of this questionnaire was approved in previous studies [14, 15]. The questionnaires content validity was evaluated by expert after translation; then two pilot studies were carried out on 120 students in Tehran. After each pilot study, the questions with any difficulty in understanding were modified. In the next step, the questionnaire validation was assessed divided by each group of questions related to a specific item (the Cronbach’s reliability coefficient >0.7). Home and school environment and psychosocial status of students were measured by 18 questions. Relationship with friends was measured by the first 5 questions; if the students gave a positive answer to these questions, it count on as a predictor of having good relationship. In the latter 5 questions, the answer A or B was taken as a predictor of having good sense in school environment [14]. Two questions measured being victim of violent behavior (being bullied) or bulling to others, while self-rated relationship difficulties with family members and friends was assessed by a 5 items. Students were asked to choose an option that indicated their status (Appendix 1: Table 4).

Height, weight, and waist circumferences were measured according to standard protocols with calibrated instruments. Weight was measured to the nearest 200 g in barefoot and lightly dressed condition. Body mass index (BMI) was calculated as weight (Kg) divided by height squared (m2) as index of obesity. Waist circumference (WC) was measured by a nonelastic tape to the nearest 0.2 cm at the end of expiration at the midpoint between the top of iliac crest and the lowest rib in standing position.

Waist- to-height ratio (WHtR), computed by dividing waist circumference (cm) to height (cm), was considered as an index of abdominal obesity [16]. We used the WHO growth curves to define BMI categories, i.e., overweight as gender-specific BMI for age of > +1 *z*-score, and obesity as gender-specific BMI for > +2 *z*-score [13]. Abdominal obesity was defined as waist to height ratio more than 0.5 [17].

The data entry staff entered data for all forms and questionnaires twice and checked for completeness and inconsistencies. The data checking process was conducted first at the district and then at the national level [13].

To assess the Screen Time (ST behaviors), the average number of hours per day that participants spent watching TV/VCDs, personal computer (PC), or electronic games [3] asked, and then the total cumulative spent time for ST was estimated. Information recorded separately for week days and weekends. According to the international ST recommendations, ST was categorized into two groups ; less than 2 h per day (Low) , and 2 h per day or more (High) [18, 19].

Through a validated questionnaire, information of past week weekly frequency of leisure time physical activity outside the school was collected. Having physical activity considered as at least 30 min duration of exercises per day which was led to heavy sweating or large increases in breathing or heart rate. Based on this provided definition, participants described their weekly PA habits through three available response choices as follow; none, 1–2 days, 3–6 days, and every day. For statistical analysis, each weekly frequency categorized into three groups; less than two times per week (Mild), two to four times a week (Moderate) and more than 4 times a week (Vigorous) [20].

The method and variables, which was used for calculating Socioeconomic status(SES) was approved previously in the Progress in the International Reading Literacy Study (PIRLS) [21]. In this study, in order to construct socioeconomic using principle component analysis (PCA) variables including parents’ education, parents’ job, possessing private car, school type (public/private), type of home (private/rented) and having personal computer in home were summarized in one main component. Using 25 % and 75 % percentiles, students were categorized into low, intermediate and high socioeconomic status.

### Statistical analyses

Continuous and categorical variables are expressed as mean (SD) and number (percentage) respectively. Logistic and linear regression was used to indicate the most important psychosocial and environmental predictors of general and abdominal obesity in Iranian adolescents. A P-value of less than 0.05 was considered as statistically significant. All statistical analyses were performed using programs available in the STATA package using survey data analysis method.

## Results

A total number of 5570 out of 5670 invited students completed the study (participation rate: 98.23 %). The average age of participants’ was 14.72 ± 2.40 years, without significant difference among girls and boys. Students consisted of 2848 (50.1 %) boys and 2835 (49.9 %) boys; 67.4 % from urban and 29.6 % from rural areas. The prevalence of overweight, generalized and abdominal obesity was 8.0 %, 8.9 % and 15.8 % respectively (Table 1).

**Table 1:** Demographic characteristics and anthropometric measures by sex in Iranian adolescents: the CASPIAN III study

		Boy	Girl	Total	*p*-value
Age (year)^a^		14.69 (2.44)	14.76 (2.37)	14.72 (2.40)	0.23
BMI (kg/m^2^)^a^		19.61(4.12)	19.23(4.06)	19.42 (4.09)	<0.001
WC (cm)^a^		67.60 (22.16)	69.86 (18.99)	68.73 (20.67)	0.001
Overweight^b^		265 (9.4)	186 (6.6)	451 (8.0)	<0.001
Obesity^b^		286 (10.1)	215 (7.7)	501 (8.9)	0.001
Abdominal obesity^b^		415 (14.7 )	473 (16.9)	888 (15.8)	0.023
Having family history of chronic diseases^b^		2277 (80.0)	2157 (76.1)	4434 (78.0)	<0.001
Screen time^b^	<2 h	568 (21.6)	479 (19.0)	1047 (20.3)	0.02
	> = 2 h	2064 (78.4)	2040 (81.0)	4104 (79.7)	
physical activity^b^	Low	1746 (69.1)	1196 (53.1)	2942 (61.5)	<0.001
	Moderate	488 (19.3)	714 (31.7)	1202 (25.1)	
	Vigorous	294 (11.6)	344 (15.3)	638 (13.3)	
Socioeconomic status^b^	Poor	868 (32.7)	870 (33.2)	1738 (33.0)	0.90
	Moderate	800 (30.1)	787(30.1)	1587 (30.1)	
	Good	987(37.2)	961(36.72)	1948 (36..9)	

^a^Data are presented as mean (SD)

^b^Data are presented as number (%)

Appendix 2: Table 5 compares mean of BMI and WC between adolescents with and without psychosocial and environmental problems. Boys which did not have best friends, spend time with their friends after school or get acceptance from them, had higher BMI than others. Only girls who did not spend time with their friends had higher BMI and WC than others. In both sexes, adolescents who had sense of pressure about doing homework did not feel good about their progress nor had difficulties in relationship with their parents had higher BMI and WC values. Girls who reported being victim of violent behaviors (being bullied), had lower BMI compared to others.

As shown in the Appendix 3: Table 6, having best friends and relationship with friends specially spending time with them after school was related to lower prevalence of overweight, obesity, and central obesity. Self-confidence only in boys led to lower obesity prevalence than adolescents lacking self-confidence.

Odds of being overweight and obese, but not abdominal obese was statistically higher in adolescents not having close friends. Moreover, adolescents who spent more time with friends after school and returning to home, had lower chance to overweight, obesity, and abdominal obesity. Lack of self-confidence increased only the risk of obesity in teens. Having difficulties in relationship with family members (parents, brothers and sisters) and friends are the predictors of overweight and obesity, too (Table 2).

**Table 2:** Odds ratios (OR) and 95 % confidence intervals [23] for home and school environment and psychosocial variables related to overweight/obesity in Iranian adolescents: the CASPIAN III study

Variables	Overweight			Obesity			Abdominal obesity		
	OR (95 % CI)			OR (95 % CI)			OR (95 % CI)		
	Model 1^a^	Model 2^b^	Model 3^c^	Model 1^a^	Model 2^b^	Model 3^c^	Model 1^a^	Model 2^b^	Model 3^c^
To have best friends (yes/no)	1.90† (1.21–2.98)	1.89† (1.20–2.97)	1.81‡ (1.11–2.95)	1.87† (1.21–2.89)	1.92† (1.24–2.98)	2.02† (1.27–3.21)	1.35 (0.92–1.99)	1.35 (0.91–1.99)	1.35 (0.89–2.04)
Spending time with friends after school (yes/no)	1.36† (1.12–1.66)	1.30† (1.07–1.59)	1.27‡ (1.03–1.57)	1.46* (1.21–1.75)	1.43* (1.18–1.72)	1.39* (1.14–1.70)	1.41* (1.22–1.63)	1.46* (1.26–1.69)	1.41* (1.21–1.66)
Spending time with friends after returning to home (yes/no)	1.51* (1.25–1.84)	1.43* (1.18–1.74)	1.50* (1.22–1.85)	1.36* (1.13–1.63)	1.30† (1.07–1.57)	1.28‡ (1.05–1.56)	1.22† (1.05–1.41)	1.26† (1.09–1.47)	1.24† (1.05–1.45)
Talking with friends via phone or mailing to them (yes/no)	0.74† (0.60–0.92)	0.79‡ (0.63–0.98)	0.82 (0.65–1.04)	0.93 (0.76–1.13)	0.97 (0.80–1.19)	1.08 (0.87–1.33)	0.92 (0.79–1.07)	0.90 (0.77–1.05)	0.94 (0.79–1.11)
Enjoying from being with friends (yes/no)	0.91 (0.73–1.14)	0.91 (0.73–1.14)	0.89 (0.70–1.12)	1.05 (0.85–1.29)	1.06 (0.86–1.30)	1.00 (0.80–1.24)	0.94 (0.80–1.11)	0.94 (0.80–1.10)	0.87 (0.73–1.04)
Self confidence (yes/no)	1.02 (0.82–1.27)	1.02 (0.82–1.27)	1.02 (0.81–1.30)	1.33† (1.09–1.64)	1.35† (1.10–1.65)	1.33† (1.07–1.65)	1.09 (0.93–1.29)	1.09 (0.93–1.29)	1.07 (0.90–1.28)
Sense of pressure while doing homework (no/yes)	0.87 (0.71–1.07)	0.87 (0.71–1.08)	0.86 (0.69–1.07)	0.88 (0.72–1.07)	0.93 (0.76–1.13)	0.89 (0.72–1.10)	0.99 (0.85–1.16)	0.98 (0.83–1.14)	0.99 (0.83–1.17)
Teacher thinks well about your progress (yes/no)	1.00 (0.83–1.23)	1.04 (0.85–1.27)	1.02 (0.82–1.26)	1.01 (0.84–1.22)	1.10 (0.91–1.34)	1.18 (0.96–1.45)	1.01 (0.87–1.17)	0.98 (0.84–1.14)	1.00 (0.85–1.18)
Sense about your school (yes/no)	0.89 (0.64–1.25)	0.89 (0.63–1.25)	0.77 (0.53–1.11)	1.26 (0.95–1.68)	1.29 (0.97–1.73)	1.10 (0.80–1.51)	1.04 (0.82–1.32)	1.03 (0.81–1.31)	0.95 (0.73–1.23)
Getting bully (no/yes)	0.79‡ (0.64–0.98)	0.80‡ (0.65–0.99)	0.80 (0.64–1.01)	1.07 (0.88–1.03)	1.06 (0.87–1.29)	1.03 (0.84–1.28)	1.06 (0.91–1.23)	1.05 (0.90–1.23)	1.07 (0.91–1.26)
Bully (no/yes)	0.75‡ (0.60–0.95)	0.78‡ (0.62–0.99)	0.81 (0.63–1.04)	1.11 (0.90–1.36)	1.13 (0.92–1.39)	1.13 (0.90–1.40)	0.96 (0.81–1.13)	0.95 (0.80–1.11)	1.00 (0.84–1.19)
Relationship difficulties with father (yes/no)	0.98 (0.80–1.20)	0.92 (0.75–1.13)	0.92 (0.74–1.15)	1.09 (0.89–1.32)	1.08 (0.89–1.32)	1.15 (0.93–1.43)	0.98 (0.84–1.15)	1.00 (0.86–1.17)	1.12 (0.95–1.33)
Relationship difficulties with mother (yes/no)	1.16 (0.93–1.44)	1.15 (0.92–1.44)	1.11 (0.88–1.42)	1.19 (0.96–1.46)	1.21 (0.98–1.50)	1.18 (0.94–1.49)	1.25† (1.06–1.46)	1.25† (1.06–1.48)	1.14 (0.95–1.37)
Relationship difficulties with brother(s) (yes/no)	1.11 (0.88–1.39)	1.05 (0.84–1.32)	0.99 (0.77–1.26)	1.64* (1.30–2.09)	1.58* (1.25–2.01)	1.40† (1.09–1.80)	1.13 (0.95–1.34)	1.17 (0.98–1.39)	1.07 (0.90–1.30)
Relationship difficulties with sister(s) (yes/no)	1.18 (0.96–1.45)	1.21 (0.99–1.50)	1.26‡ (1.00–1.57)	1.39† (1.13–1.70)	1.38† (1.13–1.70)	1.29‡ (1.04–1.61)	1.19‡ (1.02–1.39)	1.19‡ (1.01–1.39)	1.19‡ (1.00–1.41)
Relationship difficulties with close friend(s) (yes/no)	1.26‡ (1.00–1.59)	1.26‡ (1.00–1.60)	1.32‡ (1.04–1.69)	1.38† (1.11–1.71)	1.36† (1.09–1.68)	1.24 (0.99–1.56)	1.31† (1.10–1.56)	1.33† (1.11–1.58)	1.27‡ (1.05–1.53)

^a^Model 1: Without adjusted (crude models)

^b^Model 2: Adjusted for age and sex, living area

^c^Model 3: Additionally adjusted for family history of chronic disease, mother’s and father’s education, physical activity and SES

*p < 0.001, † *p* < 0.01, ‡ *p* < 0.05

Increment in BMI and WC with psychosocial and emotional problems was remained statistically significant after adjusting for age, sex, living area (urban or rural), and family history of chronic disease, mother’s education, father’s education, physical activity, and socioeconomic status (Table 3).

**Table 3:** B and SE for home and school environment and psychosocial variables related to BMI and WC in Iranian adolescents: the CASPIAN III study

Variables	BMI						WC					
	Model 1^a^		Model 2 ^b^		Model 3^c^		Model 1^a^		Model 2^b^		Model 3^c^	
	B	SE	B	SE	B	SE	B	SE	B	SE	B	SE
To have best friends (yes/no)	1.45*	0.32	1.08*	0.30	1.17*	0.31	2.01	1.63	1.01	1.59	1.10	1.74
Spending time with friends after school (yes/no)	0.63*	0.11	0.42*	0.10	0.33†	0.11	1.46*	0.55	1.34†	0.54	1.18‡	0.59
Spending time with friends after returning to home (yes/no)	0.49*	0.11	0.45*	0.11	0.44*	0.11	0.39	0.56	0.87	0.56	0.80	0.61
Talking with friends via phone or mailing to them (yes/no)	−0.34†	0.11	−0.33†	0.11	−0.20	0.11	0.21	0.58	−0.20	0.57	0.05	0.62
Enjoying from being with friends (yes/no)	0.25‡	0.12	0.11	0.11	0.06	0.12	−0.42	0.61	−0.83	0.60	−1.10	0.65
Self confidence (yes/no)	0.34†	0.13	0.27‡	0.12	0.28‡	0.12	−0.03	0.63	−0.24	0.61	−0.42	0.66
Sense of pressure while doing homework (no/yes)	0.55*	0.12	−0.05	0.11	−0.11	0.12	2.07*	0.59	0.24	0.59	0.13	0.64
Teacher thinks well about your progress (yes/no)	0.91*	0.11	0.16	0.11	0.22‡	0.11	2.80*	0.56	0.41	0.56	0.43	0.61
Sense about your school (yes/no)	0.50†	0.18	0.17	0.17	0.04	0.18	0.25	0.93	−0.68	0.91	−1.01	1.0
Getting bully (no/yes)	−0.46*	0.12	−0.07	0.11	−0.09	0.11	−1.02	0.59	−0.08	0.57	0.006	0.62
Bully (no/yes)	−0.24	0.12	−0.04	0.11	−0.02	0.12	0.45	0.63	0.71	0.61	0.86	0.67
Relationship difficulties with father (yes/no)	0.72*	0.12	0.10	0.11	0.15	0.11	0.41	0.60	−0.90	0.60	−0.71	0.65
Relationship difficulties with mother (yes/no)	0.89*	0.13	0.40*	0.12	0.37†	0.13	1.81†	0.66	0.54	0.65	0.34	0.72
Relationship difficulties with brother(s) (yes/no)	0.39†	0.13	0.39†	0.12	0.22	0.12	0.55	0.67	1.05	0.66	0.74	0.72
Relationship difficulties with sister(s) (yes/no)	−0.02	0.12	0.40*	0.11	0.33†	0.11	1.03	0.62	1.99*	0.61	1.92†	0.66
Relationship difficulties with close friend(s) (yes/no)	−0.06	0.13	0.35†	0.12	0.32‡	0.13	0.46	0.74	1.59‡	0.73	1.55‡	0.79

^a^Model 1: Without adjusted (crude models)

^b^Model 2: Adjusted for age and sex, living area

^c^Model 3: Additionally adjusted for family history of chronic disease, mother’s and father’s education, physical activity and SES

## Discussion

In this study we found that some psychosocial problems such as lack of self-confidence and proper communication with family members, friends and teachers are related to overweight, general and abdominal obesity in adolescents; however, the direction of this relationship is not very clear.

A review by Nieman revealed that overweight children and adolescents may experience deleterious psychosocial sequelae, including depression, teasing, social isolation and discrimination, diminished self-esteem, behavioural problems, dissatisfaction with body-image, and reduced quality of life [12]. Based on previous studies obesity was associated with body image dissatisfaction in boys and girls [22]. Caccavale et al. [23] found that the prevalence of overweight/obesity was higher in boys than girls but reporting body image dissatisfaction in girls is more than boys. Also the relationship between weight status and body image was moderated by social engagement only for girl adolescents. In boys, regardless of their weight status, social engagement was associated with more body image satisfaction. The gender difference may be related to the fact that girls are more likely to use social comparisons that can increase body dissatisfaction and incorrect weight-control methods in adolescent girls [24].

Most of overweight or obese adolescents may be struggling with poor body image. Obesity and poor body image are main risk factors for low self-confidence and depression, and can lead to psychosocial problems [25]. Also eating disorders may occur, which can increase the risk for weight gain [26].

Friedman et al. reported that body image satisfaction partially mediated the relationship between degree of overweight and depression/self-esteem [27]. Other study stated that body image might explain the poor self-esteem of overweight girl adolescents [28]. In a study on Turkish adolescents, body dissatisfaction was associated with poor self-esteem and depression. Also being overweight was associated with poor self-esteem, but not with depression. This study revealed that actual weight was not associated with poor self-esteem and depression. So they suggest that psychological well-being is more related to body satisfaction than actual weight status [29].

Another study revealed that early onset of obesity has a negative effect on body image in adult life; it means that emerging the obesity in younger people increases the risk of body dissatisfaction, which can impair self-esteem [30].

The results about the relationship between overweight/obesity and psychopathology in different studies are inconsistent. In a study on school-aged adolescents in Ghana and Uganda a psychosocial factor (e.g. loneliness) was significantly associated with overweight or obesity [31]. In a longitudinal study Goodman and his colleague found that the risk for persistence and developing obesity during adolescence is increased at depressed adolescents [32].

In Stice’s study body image dissatisfaction was a significant determinant in predicting for developing adolescent girls depression later [33].

Recent studies revealed that weight criticism and body dissatisfaction caused to lower participation in vigorous physical activity, among overweight girls [34]. Eating behaviors disorders and unhealthy weight control methods among overweight adolescents is more often than others [35].

The associations of overweight, body image, and depressive symptoms may be related to the influence of pubertal development in adolescents. It means that pubertal timing and velocity of transition is related to body fat accumulation, which may be related to different experience of body image concerns and risk of depression. In pubertal development, both growth and sex hormones cause to increase in fat mass accumulation and because of this additional body fat, puberty is related to enhancing the risk of body dissatisfaction [36].

Depression was negatively associated with breakfast consumption behavior in Indian adolescents [37]. The findings of Indian study are in line with similar studies carried out in developed countries which suggested that depression is negatively associated with healthy dietary behaviors such as consuming breakfast, lunch and dinner [38]. A Meta-analytic study found that there was no significant relationship between obesity and depression in the population [39].

Ozmen et al., in their study revealed that overweight based on BMI had no effect on self-esteem and depression [39]. Also, Kim and his colleague stated that weight status based on BMI was not a good predictor for the level of self-esteem and depression [40]. The level of self-esteem and anxiety of obese adolescents did not differ from normal weight ones [41]. In a clinical population, a higher ratio of depression, behavioral problems, poor self-esteem was reported [42]. A literature review study revealed that the relationship between self-esteem and obesity was unclear whether self-esteem was consistently related to obesity [43]. So, there are multiple patterns for association between overweight/obesity and psychosocial risk factors in adolescents. It means that there are some other factors which can mediate the relationship between obesity and psychosocial distress.

In a study carried out on Portuguese adolescents, there was no significant difference between the number of close friends and obesity whereas potential social isolation was associated with overweight among the subjects. Also this study revealed that making new friends was more difficult for obese and overweight adolescents in comparison to non-obese and non-overweight peers [1]. These overweight children and adolescents with co-morbid psychiatric problems and issues with socialization and self-esteem need special interventions [44].

### Study limitations and strengths

This study provides valuable information about weight status and some psychosocial factors of adolescents living in Iran. However, this study has some limitations. We didn’t assess household structure (child living with: both parent, mother only, father only, and grandparents only) regarding the risk of overweight and obesity. Therefore, more studies are needed to further explore the role of household structure in the context of parental influences on weight status and some psychosocial factors.

## Conclusion

Our findings may greatly contribute to better understanding the association of weight disorders and some psychosocial factors adolescents. The high prevalence of adolescent boys (33.9 %) and girls (27.4 %) who were overweight/ obese in this nationally representative sample, along with the consistent association between overweight/obesity and psychosocial variables highlight the need for concurrent interventions for two problems in adolescents.

## Abbreviations

CASPIAN, **C**hildhood and **A**dolescence **S**urveillance and **P**revent**I**on of **A**dult **N**on communicable disease

## Appendix

### Appendix 1

**Table 4:** List of questions to screen psychosocial factors according to Global School-based student Health Survey (GSHS) questionnaires

Question	Response
How many close friends do you have?	1. 0 (considered as No) 2. 1 (considered as Yes) 3. 2 (considered as Yes) 4. 3 or more (considered as Yes)
During the past 30 days, how often did you spend time with friends after school?	1. Never (considered as No) 2. 1–2 times (considered as Yes) 3. 2–3 times (considered as Yes) 4. 4 times or more (considered as Yes)
During the past 30 days, how often did you spend time with friends after returning to home?	1. Never (considered as No) 2. 1–2 times (considered as Yes) 3. 2–3 times (considered as Yes) 4. 4 times or more (considered as Yes)
During the past 30 days, how often did you talk with friends via phone or mailing to them?	1. Never (considered as No) 2. 1–2 times (considered as Yes) 3. 2–3 times (considered as Yes) 4. 4 times or more (considered as Yes)
During the past 30 days, how often did you enjoy from being with friends?	1. Never (considered as No) 2. 1–2 times (considered as Yes) 3. 2–3 times (considered as Yes) 4. 4 times or more (considered as Yes)
Feel accepted was assessed by agreement with this statement: “other students accept me as I am”	1. Strongly agree (considered as Yes) 2. Agree (considered as Yes) 3. Neither agree nor disagree (considered as No) 4. Disagree (considered as No) 5. Strongly disagree
During the past 3 months, how many times you were bullied?	1. Never (considered as No) 2. 1–2 times (considered as Yes) 3. 2–3 times (considered as Yes) 4. 4 times or more (considered as Yes)
During the past 3 months, how many times you got bullied?	1. Never (considered as No) 2. 1–2 times (considered as Yes) 3. 2–3 times (considered as Yes) 4. 4 times or more (considered as Yes)
Which of your parent are you most comfortable with sharing your problem?	1. None of them (Considered as No) 2. One of them (Considered as Yes) 3. Both of them (Considered as Yes)
During your life, how many times have you got into trouble with your family or friends?	1. None of them (Considered as No) 2. Father (Considered as Yes) 3. Mother (Considered as Yes) 4. Brother (Considered as Yes) 5. Sister (Considered as Yes) 6. Close friends (Considered as Yes)
Do you like your school at present?	1. I like it very much (considered as Yes) 2. I like it a bit (considered as Yes) 3. I don’t like it very much (considered as No) 4. I don’t like it at all (considered as No)
What do your teachers think about your performance in comparison with your classmates?	1. Very good (considered as good) 2. Good(considered as good) 3. average (considered as not good) 4. below average (considered as not good)
During the past 12 months, how often have you felt lonely?	1. Never (Considered as No) 2. Rarely (Considered as No) 3. Sometimes (Considered as Yes) 4. Most of the time (Considered as Yes) 5. Always (Considered as Yes)
During the past 30 days, how often did your parents or guardians expect too much of you (for example, to do better in school or be a better person)?	1. Never (considered as No) 2. Rarely (considered as No) 3. Sometimes (considered as Yes) 4. Most of the time (considered as Yes) 5. Always (considered as Yes)
Did you enjoy spending time with your parents or guardians?	1. Strongly agree (considered as Yes) 2. Agree (considered as Yes) 3. Neither agree nor disagree (considered as No) 4. Disagree (considered as No) Strongly disagree

### Appendix 2

**Table 5:** Mean and Standard deviation of BMI and waist circumference related to home and school environment and psychosocial variables in Iranian adolescents: the CASPIAN III study

Variables		BMI (kg/m^2^)		WC (cm)	
		Boy	Girl	Boy	Girl
		Mean (SD)	Mean (SD)	Mean (SD)	Mean (SD)
To have best friends	Yes	19.54(4.09)*	19.21(4.07)	67.54(22.45)	69.79(19.17)
	No	21.48(4.47)	20.10(4.05)	69.12(10.25)	72.45(11.92)
Spending time with friends after school	Yes	19.20(3.99)*	19.09(3.92)†	66.66(22.43)†	69.15(17.43)†
	No	19.97(4.21)	19.48(4.28)	68.46(22.07)	71.04(21.29)
Spending time with friends after returning to home	Yes	19.31(3.96)*	19.18(4.06)	66.83(21.27)	69.93(21.22)
	No	19.95(4.28)	19.36(4.08)	68.47(23.25)	69.81(12.14)
Talking with friends via phone or mailing to them	Yes	19.65(4.04)	19.38(4.04)	67.51(19.09)	70.03(11.97)
	No	19.49(4.32)	18.98(4.03)	67.82(28.72)	69.57(26.05)
Enjoying from being with friends	Yes	19.55(4.08)	19.17(4.05)	67.70(25.11)	70.01(21.15)
	No	19.79(4.23)	19.42(4.10)	67.37(11.35)	69.48(11.65)
Self confidence	Yes	19.47(4.06)‡	19.18(4.04)	67.54(25.12)	69.89(20.99)
	No	19.95(4.24)	19.37(4.12)	67.71(10.24)	69.66(11.67)
Sense of pressure while doing homework	Yes	19.19(4.20)*	18.89(4.09)‡	65.80(11.88)‡	68.84(22.14)
	No	19.81(4.07)	19.38(4.04)	68.44(25.53)	70.26(17.48)
Teacher thinks well about your progress	Yes	19.25(4.10)*	18.83(3.94)*	66.63(22.85)‡	68.61(18.55)*
	No	20.19(4.08)	19.75(4.17)	69.19(21.04)	71.43(19.54)
Sense about your school	Yes	19.62(4.11)†	19.25(4.03)	67.72(23.42)	70.06(19.93)
	No	20.27(4.19)	19.60(4.48)	68.34(11.70)	69.98(11.73)
Getting Bully	No	19.66(4.05)	19.49(4.14)*	67.74(23.76)	70.54(17.34)
	Yes	19.49(4.28)	18.81(3.90)	67.25(18.07)	68.72(21.73)
Bully	No	19.63(4.18)	19.34(4.06)	67.53(19.09)	69.83(11.85)
	Yes	19.60(3.97)	18.99(4.07)	67.96(30.72)	69.89(29.21)
Relationship difficulties with Father	No	19.15(4.11)*	19.03(3.97)*	67.39(30.61)	69.48(22.44)
	Yes	19.96(4.09)	19.55(4.25)	67.74(10.87)	70.81(12.10)
Relationship difficulties with Mother	No	19.31(4.12)*	18.96(4.00)*	67.10(25.95)	69.16(21.40)‡
	Yes	20.13(4.03)	19.89(4.24)	68.44(11.33)	71.80(12.01)
Relationship difficulties with Brother(s)	No	19.24(3.98)	19.01(3.85)	66.98(19.67)	69.32(21.00)
	Yes	19.64(4.12)	19.29(4.12)	67.74(24.53)	70.37(19.30)
Relationship difficulties with Sister(s)	No	19.64(3.96)	19.08(3.94)	67.26(11.30)	69.46(11.61)
	Yes	19.48(4.24)	19.27(4.10)	68.07(31.12)	70.38(24.60)
Relationship difficulties with close friend(s)	No	19.60(4.00)	19.13(3.96)	67.32(21.69)	69.66(18.78)
	Yes	19.30(4.28)	19.31(4.34)	67.67(29.33)	70.17(24.39)

**P* < 0.001, † *p* < 0.05, ‡ *p* < 0.01

### Appendix 3

**Table 6:** Prevalence of overweight, obesity and central obesity based on home and school environment and psychosocial variables in Iranian adolescents: the CASPIAN III study

Variables		Overweight		General obesity		Abdominal obesity	
		Boy	Girl	Boy	Girl	Boy	Girl
To have best friends	Yes	254(9.31)	174(6.41)*	267(9.79)*	207(7.63)	397(14.53)	454(16.72)
	No	11(12.50)	12(15.58)	18(20.45)	7(9.09)	16(18.18)	17(22.08)
Spending time with friends after school	Yes	106(8.17)	101(5.96)	110(8.48)†	115(6.78)‡	163(12.56)†	245(14.47)*
	No	154(10.28)	83(7.71)	175(11.68)	98(9.10)	247(16.46)	224(20.74)
Spending time with friends after returning to home	Yes	124(8.31)‡	111(5.67)†	129(8.65)†	145(7.41)	197(13.19)‡	313(15.99)‡
	No	140(10.67)	73(8.87)	156(11.89)	68(8.26)	216(16.43)	157(19.05)
Talking with friends via phone or mailing to them	Yes	194(9.55)	126(7.65)†	207(10.19)	125(7.59)	289(14.22)	302(18.33)†
	No	70(8.93)	58(5.10)	77(9.82)	85(7.48)	124(15.78)	164(14.42)
Enjoying from being with friends	Yes	198(9.68)	136(6.37)	205(10.02)	153(7.57)	301(14.71)	350(17.32)
	No	66(8.60)	50(6.53)	80(10.43)	61(7.96)	112(14.55)	122(15.89)
Self confidence	Yes	192(9.26)	141(6.84)	192(9.26)‡	147(7.13)	296(14.26)	341(16.54)
	No	73(10.07)	45(6.28)	88(12.14)	66(9.21)	114(15.68)	126(17.57)
Sense of pressure while doing homework	No	91(10.33)	60(7.10)	97(11.01)	69(8.17)	125(14.11)	149(17.67)
	Yes	173(8.95)	125(6.46)	189(9.78)	143(7.39)	290(15.01)	319(16.47)
Teacher thinks well about your progress	Yes	158(9.00)	109(6.87)	180(10.26)	114(7.18)	249(14.16)	276(17.36)
	No	106(10.08)	75(6.28)	101(9.60)	99(8.29)	163(15.49)	192(16.11)
Sense about your school	Yes	226(9.38)	155(6.46)	240(9.96)	179(7.46)	344(14.26)	407(16.97)
	No	22(7.72)	18(6.57)	35(12.28)	25(9.12)	51(17.89)	39(14.23)
Getting bully	No	192(9.57)	131(7.32)	186(9.44)	142(7.93)	275(13.93)	308(17.20)
	Yes	72(8.60)	54(5.45)	97(11.59)	73(7.37)	135(16.13)	162(16.36)
Bully	No	208(9.71)	133(7.02)	212(9.89)	140(7.39)	319(14.86)	325(17.13)
	Yes	51(7.97)	47(5.43)	71(11.09)	73(8.43)	90(14.04)	142(16.40)
Relationship difficulties with Father	Yes	121(9.43)	119(7.18)	127(9.90)	124(7.48)	183(14.25)	280(16.91)
	No	126(9.65)	49(5.59)	131(10.03)	70(7.99)	192(14.67)	148(16.84)
Relationship difficulties with Mother	Yes	163(8.74)	125(6.53)	184(9.87)	133(6.95)‡	250(13.38)	302(15.75)‡
	No	80(10.24)	44(6.89)	77(9.86)	62(9.70)	124(15.88)	125(19.56)
Relationship difficulties with Brother(s)	Yes	59(9.25)	56(6.18)	47(7.37)‡	48(5.30)†	94(14.73)	124(13.70)*
	No	168(9.25)	99(6.87)	192(10.57)	125(8.67)	248(13.63)	264(18.27)
Relationship difficulties with sister(s)	Yes	111(8.45)	67(6.32)	108(8.22)†	65(6.13)	179(13.62)	158(14.91)‡
	No	124(10.21)	97(7.40)	141(11.61)	107(8.17)	178(14.63)	238(18.14)
Relationship difficulties with close friend(s)	Yes	114(8.28)	76(5.89)‡	127(9.23)	89(6.90)‡	176(12.77)	194(15.00)†
	No	71(9.48)	61(8.19)	89(11.88)	73(9.80)	114(15.18)	147(19.71)

Data are presented as *N*(%)

**p* < 0.001, † *p* < 0.01, ‡ *p* < 0.05
